# Supplementary material for: Monitoring peripheral perfusion in sepsis associated acute kidney injury: Analysis of mortality
Source: PLoS One. 2020 Oct 14;15(10):e0239770. doi: 10.1371/journal.pone.0239770 (PMC7556522; doi:10.1371/journal.pone.0239770)
Supplement: S1 Table — (PDF) [file pone.0239770.s001.pdf]

S1 Table. The demographic, clinical, and hemodynamic of septic patients after fluid resuscitation.

**Parameters**	**All patients n =141**	**Non-SA-AKI group n = 28**	**SA-AKI group n =113**	**P-value**
**Clinical**				
Age, mean (SD), y	55 (17)	50 (18)	57 (17)	0.09
Sex, n (%)				0.67
Men	82 (58.2)	15 (53.6)	67 (59.3)	
Women	59 (41.8)	13 (46.4)	46 (40.7)	
Comorbidities, No. (%)				
Diabetes mellitus	24 (17)	4 (14.3)	20 (17.7)	0.78
Hypertension	49 (34.8)	6 (21.4)	43 (38.1)	0.12
Chronic kidney disease	13 (9.2)	0 (0)	13 (11.5)	0.07
Heart failure	21 (14.9)	2 (7.1)	19 (16.8)	0.25
Liver failure	8 (5.7)	3 (10.7)	5 (4.4)	0.19
Cerebral vascular disease	7 (5)	2 (7.1)	5 (4.4)	0.63
Chronic pulmonary disease	23 (16.3)	2 (7.1)	21 (18.6)	0.25
Cancer	27 (19.1)	4 (14.3)	23 (20.4)	0.60
Immunosuppression	28 (19.9)	8 (28.6)	20 (17.7)	0.20
**Source of infection,** No. (%)				
Respiratory	66 (46.8)	15 (53.6)	51 (45.1)	0.53
Abdominal	38 (27.7)	4 (14.3)	34 (30.1)	0.10
Urinary	16 (11.3)	1 (3.6)	15 (13.3)	0.19
Others	21 (15.6)	8 (28.6)	14 (12.4)	0.04*
**Any microorganism in cultures** No. (%)	76 (53.9)	12 (42.9)	64 (56.6)	0.21
**Confirmed bloodstream infection,** No. (%)	42 (29.8)	9 (32.1)	33 (29.2)	0.82
**Scores and Biomarkers at ICU admission**				
SOFA score, mean (SD)[a]	10 (4)	8 (3)	11 (4)	0.00**
APACHE II score, mean (SD)[b]	24 (8)	18 (6)	25 (7)	0.00**
CRP, mean (SD), mg/dl	16 (11)	16(8)	17 (11)	0.73
Procalcitonin, No./median (IQR), ng/ml	105/3.4 (1-17)	20/2.3 (0.9-15)	85/ 4 (1.5-18)	0.11
Creatinine, median (IQR), µmol/L	124 (71-248)	71 (53-88)	150 (88-292)	0.00**
Urea nitrogen serum, median (IQR), mmol/L	30 (15-41)	16 (10-23)	29 (16-44)	0.00**
**Hemodynamic data after resuscitation**				
PAM, mean (SD), mmHg	86 (20)	87 (15)	86 (21)	0.69
Heart Rate, mean (SD), /min	95 (22)	93 (16)	96 (24)	0.56
$ScvO_2$, No. / median (IQR), %	76/ 73 (67-78)	12/ 72 (62-78)	64/ 72 (68-78)	0.55
Pv-aCO_2, No. / mean (SD), mmHg	70/ 7 (4)	9/ 5 (3)	61/ 7 (4)	0.20
Arterial lactate, median (IQR), mmol/L	2.0 (1.4-2.9)	1.7 (1.2-2)	2.1 (1.5-3)	0.01**
Urine Output, median (IQR), ml/kg/h	0.6 (0.29-1.1)	0.7 (0.6-1.3)	0.6 (0.2-1.1)	0.02*
**Vasoactive drugs use,** No. (%)	103 (73)	14 (50)	89 (79)	0.00**
**Noradreline dose,** median (IQR), µg/kg/min	0.3 (0.1-0.5)	0.3 (0.1-0.4)	0.2 (0.1-0.6)	0.66
**Vasopressin use,** No. (%)	30 (21.3)	3 (10.7)	27 (23.9)	0.20

Legend 1:** $p < 0.01$; * $p < 0.05$.

Footnote 1. Abbreviations: APACHE, Acute Physiology, and Chronic Health Evaluation; SOFA, Sequential Organ Failure Assessment; CRP, C- reactive protein; MAP, mean arterial pressure; $ScvO_2$, central venous oxygen saturation; $Pv\text{-}aCO_2$, venous to arterial carbon dioxide difference; SA-AKI: Sepsis associated Acute Kidney Injury.

[a] Range, 0 to 24: higher scores are associated with the intensity of organ dysfunction and a higher risk of in-hospital death. (22)

[b] Range, 0 to 71: higher scores are associated with the intensity of illness and a higher risk of in-hospital death. (22)
