# Supplementary material for: Monitoring peripheral perfusion in sepsis associated acute kidney injury: Analysis of mortality
Source: PLoS One. 2020 Oct 14;15(10):e0239770. doi: 10.1371/journal.pone.0239770 (PMC7556522; doi:10.1371/journal.pone.0239770)
Supplement: S2 Table — (PDF) [file pone.0239770.s002.pdf]

S2 Table. The demographic, clinical and hemodynamic of SA-AKI group after fluid resuscitation.

**Parameters**	**SA-AKI group n =113**	**Survivors n=51**	**Nonsurvivors n = 62**	**P-value**
Clinical				
Age, mean (SD), y	57 (17)	53 (16)	59 (18)	0.07
Sex, n (%)				0.12
Men	67 (59.3)	26 (51)	41 (66.1)	
Women	46 (40.7)	25 (49)	21 (33.9)	
Comorbidities, No. (%)				
Diabetes mellitus	20 (17.7)	7 (13.7)	13 (21)	0.34
Hypertension	43 (38.1)	14 (27.5)	29 (46.8)	0.05
Chronic kidney disease	13 (11.5)	3 (5.9)	10 (16.1)	0.14
Heart failure	19 (16.8)	9 (17.6)	10 (16.1)	1.00
Liver failure	5 (4.4)	2 (3.9)	3 (4.8)	1.00
Cerebral vascular disease	5 (4.4)	2 (3.9)	3 (4.8)	1.00
Chronic pulmonary disease	21 (18.6)	8 (15.7)	13 (21)	0.63
Cancer	23 (20.4)	7 (13.7)	16 (25.8)	0.16
Immunosuppression	20 (17.7)	10 (19.6)	10 (16.1)	0.63
Source of infection, No. (%)				
Respiratory	51 (45.1)	20 (39.2)	31 (50)	0.26
Abdominal	34 (30.1)	15 (29.4)	20 (32.3)	0.84
Urinary	15 (13.3)	10 (19.6)	5 (8.1)	0.10
Others	14 (12.4)	6 (11.8)	8 (12.9)	1.00
Any microorganism in cultures No. (%)	64 (56.6)	31(60.8)	33 (53.2)	0.45
Confirmed bloodstream infection, No. (%)	33 (29.2)	10 (19.6)	23 (37.1)	0.06
Scores and Biomarkers at ICU admission				
SOFA score, mean (SD)a	11 (4)	9 (4)	12 (3)	0.00**
APACHE II score, mean (SD)b	25 (7)	21 (7)	28 (6)	0.00**
CRP, mean (SD), mg/dl	17 (11)	17 (11)	17 (11)	0.89
Procalcitonin, No./median (IQR), ng/ml	85/ 4 (1.5-18)	36/ 2.8 (1.5-13)	49/ 5.5 (1.5-22)	0.37
Creatinine, median (IQR), µmol/L	150 (88-292)	123.7 (75-252)	190 (88-309)	0.05
Urea nitrogen serum, median (IQR), mmol/L	29 (16-44)	21.0 (14-40)	34 (20-47)	0.00**
Hemodynamic data after resuscitation				
PAM, mean (SD), mmHg	86 (21)	86 (15)	86 (25)	0.93
Heart Rate, mean (SD), /min	96 (24)	91 (22)	101 (24)	0.03*
ScvO2, No. / median (IQR), %	64/ 72 (68-78)	20/74 (71-76)	44/72 (66-79)	0.34
Pv-aCO2, No. / mean (SD), mmHg	61/ 7 (4)	20/6 (3)	42/7 (4)	0.06
Arterial lactate, median (IQR), mmol/L	2.1 (1.5-3)	1.9 (1.4-2.7)	2.3 (1.7-3.5)	0.06
Urine Output, median (IQR), ml/kg/h	0.6 (0.2-1.1)	0.6 (0.3-1.3)	0.6 (0.2-1.1)	0.32
Vasoactive drugs use, No. (%)	89 (79)	36 (40.4)	53 (59.3)	0.07
Noradrenaline dose, median (IQR), µg/kg/min	0.2 (0.1-0.6)	0.2 (0.1-0.2)	0.4 (0.2-0.7)	0.00**
Vasopressin use, No. (%)	27 (23.9)	4 (7.8)	23 (37.1)	0.00**

In-hospital Hemodialysis, No. (%)	11 (9.7)	2 (3.9)	9 (14.5)	0.11

Legend 2:** $p < 0.01$; * $p < 0.05$.

Footnote 2. Abbreviations: APACHE, Acute Physiology, and Chronic Health Evaluation; SOFA, Sequential Organ Failure Assessment; CRP, C- reactive protein; MAP, mean arterial pressure; $ScvO_2$, central venous oxygen saturation; Pv-aCO_2, venous to arterial carbon dioxide difference; SA-AKI: Sepsis associated Acute Kidney Injury.

[a] Range, 0 to 24: higher scores are associated with the intensity of organ dysfunction and a higher risk of in-hospital death. (22)

[b] Range, 0 to 71: higher scores are associated with the severity of illness and a higher risk of in-hospital mortality. (22)
