# Supplementary material for: Monitoring peripheral perfusion in sepsis associated acute kidney injury: Analysis of mortality
Source: PLoS One. 2020 Oct 14;15(10):e0239770. doi: 10.1371/journal.pone.0239770 (PMC7556522; doi:10.1371/journal.pone.0239770)
Supplement: S3 Table — (PDF) [file pone.0239770.s003.pdf]

S3 Table. Prevalence of Peripheral hypoperfusion in first 24 hours between groups

Method	No AKI	AKI diagnosis first 24 h	AKI diagnosis after 24 h
PI < 1.4	8/28 (28%)	33/69 (47%)	18/44 (40%)
CRT ≥ 3 s	6/28 (21%)	37/69 (53%)*	24/44 (54%)*

Legend 3: (A) No AKI vs AKI diagnosis first 24 h * $p < 0.05$; (B) No AKI vs Aki diagnosis after 24 h $p < 0.05$; (C) AKI diagnosis first 24 h vs Aki diagnosis after 24 h not significant. Comparative analyses of the prevalence of peripheral hypoperfusion between groups were performed using Fisher's exact test. The Bonferroni test was subsequently added for correction due to multiple comparisons.
