# Supplementary material for: Monitoring peripheral perfusion in sepsis associated acute kidney injury: Analysis of mortality
Source: PLoS One. 2020 Oct 14;15(10):e0239770. doi: 10.1371/journal.pone.0239770 (PMC7556522; doi:10.1371/journal.pone.0239770)
Supplement: Attachment — Submitted filename: Response to Reviewers.docx [file pone.0239770.s006.docx]

**Response to Reviewers**

We would like to extend our gratitude to the reviewers for their detailed and instructive comments, and we hope that we have sufficiently addressed the concerns raised. We believe the revised manuscript is much clearer and stronger due to the comments provided. Listed below are changes made in response to reviewers’ comments.

**ACADEMIC EDITOR**

1. Please ensure that your manuscript meets PLOS ONE's style requirements, including those for file naming. The PLOS ONE style templates can be found at

<https://journals.plos.org/plosone/s/file?id=wjVg/PLOSOne_formatting_sample_main_body.pdf> and

<https://journals.plos.org/plosone/s/file?id=ba62/PLOSOne_formatting_sample_title_authors_affiliations.pdf>

**Response:** Our manuscript meted PLOS ONE's style requirements.

2.Thank you for stating the following financial disclosure:

 [The funders had no role in study design, data collection, and analysis, decision to publish, or preparation of the manuscript.].

At this time, please address the following queries:

a. Please clarify the sources of funding (financial or material support) for your study. List the grants or organizations that supported your study, including funding received from your institution.
b. State what role the funders took in the study. If the funders had no role in your study, please state: “The funders had no role in study design, data collection, and analysis, decision to publish, or preparation of the manuscript.”
c. If any authors received a salary from any of your funders, please state which authors and which funders.
d. If you did not receive any funding for this study, please state: “The authors received no specific funding for this work.”

Please include your amended statements within your cover letter; we will change the online submission form on your behalf.

**Response:** “The authors received no specific funding for this work.”

We included this sentence in the cover letter.

3.Thank you for including your ethics statement: 'All participants or their legal representatives provided written informed consent, and the Research Ethics Committee approved the research of the Federal University of Paraná (protocol: 3.142.086/2019).'

(a) Please amend your current ethics statement to include the full name of the ethics committee/institutional review board(s) that approved your specific study.

(b) Once you have amended this/these statement(s) in the Methods section of the manuscript, please add the same text to the “Ethics Statement” field of the submission form (via “Edit Submission”).

For additional information about PLOS ONE ethical requirements for human subjects research, please refer to <http://journals.plos.org/plosone/s/submission-guidelines#loc-human-subjects-research>.

**Response:** The full name of the ethics committee/institutional that approved our study is “Human Research Ethics Committee” of the Hospital de Clínicas, Federal University of Paraná.

We amended this statement in the Methods section to make it clearer (p. 4) and added the same text to the “Ethics Statement” field of the submission form.

**REVIEWER #1**

1. "To the best of our knowledge, this is the first study to comprehensively and accurately address the importance of monitoring peripheral perfusion in Sepsis associated AKI (SA-AKI)."---this statement is not true because peripheral perfusion has been extensively explored. I suggest rephrasing this sentence to a more focused area with novelty.

**Response:** We have come to agree with the reviewer that peripheral perfusion has been extensively explored, as demonstrated in our recent article review that identified more than 20 related studies (1). However, the literature is scarce regarding the importance of this type of monitoring for each kind of organ dysfunction associated with sepsis since the various dysfunctions imply clinical evaluation and prognostic heterogeneity. After careful consideration, to meet reviewers question, we rephrased this sentence to: "Although the peripheral perfusion has been extensively studied in non-selected septic patients, to the best of our knowledge, the potential role of clinical assessment of peripheral perfusion as a possible tool for SA-AKI management has not been established." (p. 4).

2. This is a cohort study, not a case-control study; so, you need to explicitly declare this.

**Response:** This has now been corrected (p. 2, 4).

3. The time window for the PI and CRT measurement should be explicitly declared; and its relationship to the AKI assessment should also be clarified. This is important for causal inference.

**Response:** It is well known that dynamic circulatory changes attributed to sepsis lead to the different time-related clinical significance of peripheral perfusion values.(1) Our study selected the same assessment time in all groups (SA-AKI and non-SA-AKI groups) to reduce the comparative bias. Our objective was to analyze a relatively early stage of sepsis associated with a similar hemodynamic status. The moment for assessment of peripheral perfusion was after fluid resuscitation between 6 and 24 hours of sepsis diagnosis. We revised the Methods section to make it more transparent about how the analysis was conducted (p. 6).

Our First outcome was the prevalence of peripheral hypoperfusion in SA-AKI patients. In this sense, we cannot make a causal inference about this outcome (AKI). In this first study design, we can only formulate a hypothesis of possible association as described in the Discussion section (p. 12 and 13).

4. Details of the Linear Model Mixed Effects test should be clarified. For example, did you allow the slope to differ between individuals? or random-effects were just allowed for the intercept term? How did you specify random-effects and fixed-effect terms?

**Response:** A linear mixed-effects model with random intercept and slope was used to test hypotheses related to the group (survivors and nonsurvivors), time (first, second, and third days), and interaction between time and group. Group was considered a fixed effect and patient as a random effect.

We revised the Analytical approach to make it more straightforward as to how the analysis was conducted. Please see the Methods section (p. 8).

5. When you identify independent predictors for AKI, you must clarify the temporal relationship between outcome and predictors. The outcome (AKI) may be present on ICU admission.

**Response:** Thank you for bringing this critical issue to our attention. In our study, Acute kidney injury was defined according to Kidney Disease Improving Global Outcomes (KDIGO) based on serum creatinine and urine output criteria (2). Unfortunately, the existing criteria are advantageous and widely validated, it has limitations like the limit of time when starting the damage. Independently of the number of SA-AKI patients present on ICU admission, the peripheral perfusion assessment was established over the first 24 hours. In contrast, the AKI diagnosis was made over the first seven days of sepsis diagnosis. In response to the reviewer's requirement, we have made a new analysis where the patients were divided by groups: Patients with no AKI; Patients with AKI diagnosis over the first 24 hours; and Patients with AKI diagnosis after 24 hours. As shown in S3 Table, no prevalence differences of peripheral hypoperfusion were observed between patients who had AKI diagnosis over the first 24 hours and Patients with AKI diagnosis after 24 hours. This result suggests that at least over the first seven days, the time of AKI diagnosis doesn't seem to interfere in peripheral perfusion existence.

Comparative analyses of the prevalence of peripheral hypoperfusion between SA-AKI diagnosis over the first 24 h, AKI diagnosis after 24 h and non-SA-AKI groups with predefined cutoffs for abnormal perfusion (CRT ≥ 3 seconds and PI < 1.4) were performed using Fisher's exact test. The Holm Bonferroni test was subsequently added for correction due to multiple comparisons.

**S3 Table. Prevalence of Peripheral hypoperfusion in first 24 hours between groups.**

| Method | No AKI | AKI diagnosis first 24 h | AKI diagnosis after 24 h |
| --- | --- | --- | --- |
| PI < 1.4 | 8/28 (28%) | 33/69 (47%) | 18/44 (40%) |
| CRT ≥ 3 s | 6/28 (21%) | 37/69 (53%)* | 24/44 (54%)* |

(A) No AKI versus (vs) AKI diagnosis first 24 h * p < 0.05; (B) No AKI vs Aki diagnosis after 24 h p < 0.05; (C) AKI diagnosis first 24 h vs Aki diagnosis after 24 h not significant. Comparative analyses of the prevalence of peripheral hypoperfusion between groups were performed using Fisher's exact test. The Bonferroni test was subsequently added for correction due to multiple comparisons.

We added these analyses in the Results section (p.10), and the S3 Table in the Supporting information section.

6. When you explore the association of fluid balance and PI/CRT, you need to clarify the temporal relationship in the METHOD section.

**Response:** We have added the following: “To speculate if the fluid balance could be a link factor that associates peripheral hypoperfusion with the development and prognosis of patients with SA-AKI, a correlation between the cumulative 24 hours fluid balanced were correlated with the 24 h PI values after sepsis diagnosis.”

We revised the section on misuses in the Methods section to make it clearer as to how the analysis was conducted. Please see the Methods section (p. 7).

**REVIEWER #2**

**Abstract:**
1. Could the authors shortly mention here that AKI was defined based on KDIGO on both serum creatinine and urine output?

**Response:** Thank you for raising this issue. We added this information in the Abstract section (p. 2). However, we omit the reference (KDIGO) according to instructions of Submission Guidelines by “Plos One”. This is better described in the Methods section (p. 2).

2. Numbers at the beginning of a sentence should be fully spelled.

**Response:** This has now been corrected (p. 2).

3. In the last part, it is mentioned that the presence of peripheral hypoperfusion may be an important prognostic marker. Could the authors add here for what specific outcome this prognostic value is?

**Response:** It is an important prognostic marker for mortality. This was added in the Abstract section (p. 2).

**Introduction:**
4. Could the authors elaborate either here or in the discussion about how the ´´phase of sepsis´´ may influence the made measurements? I.e. at certain points patients may have a high cardiac index with even shortened CRT before suffering from peripheral hypoperfusion and a prolonged CRT.

**Response:** The reviewer is correct. Although we have already cited this subject in the discussion section, we added an “extensive” discussion concerning this issue in the discussion section (p. 13).

5. On page 4, there are numbers 1 and 3 in superscript. Should these be references? Please clarify.

**Response:** These are references. This has now been corrected (p. 4).

6. In the last section of the introduction (and in the title), the authors mention clinical outcomes, where later the most focus is on mortality. Could this be specified or changed?

**Response:** Yes. The words “clinical outcomes” have been replaced by “mortality” term (p. 1, 2, 4). Thanks for your suggestion.

**Methods:**
7. One of the exclusion criteria is inaccessible peripheral perfusion, could the authors provide an example?

**Response:** Severe Hypothermia, Raynaud's Syndrome, Peripheral Arterial Occlusive Diseases and, Scleroderma are examples of inaccessible peripheral perfusion.

We revised the section on misuses in the Methods section to make it clearer as to how the analysis was conducted. Please see the Methods section (p. 5).

8. How were missing values handled? I.e. missing urine output data or missing pre-admission creatinine? Was a baseline eGFR assumed?

**Response:** As described in the Methods section (p. 5): “Baseline kidney function was defined as the most recent outpatient, non-emergency department serum creatinine concentration between 7 and 365 days before sepsis diagnosis”. For patients with missing output data, only serum creatinine was taken into consideration. Of 141 patients, 7 had missed output data.

9. The CRT was measured at what place? Index finger? This is important for the reference value.

**Response:** The CRT and PI assessment were made in the index finger.

Thank you for raising this issue. We added this information in the Methods section (p. 6).

10. How did the authors decide on the limit of 3 secs for the CRT to be abnormal? In the stated reference, 2.4 s is mentioned for septic shock pts and 5 for critically ill, previous papers have stated 4.5 seconds.

**Response:** The cutoff value was selected based on 2 robust studies:

1. The study by AIT-OUFELLA et al. (2014) which demonstrated that a cutoff point of 2.4 seconds could predict outcomes in 14 days with good sensitivity and specificity. (3)
2. The ANDROMEDA-SHOCK study demonstrated that a CRT cutoff point of ≤ 3 seconds showed similar effects to the standard protocol based on the decrease of serum lactate levels as a goal after hemodynamic resuscitation with fluid to reduce SOFA score after 72 hours and mortality at 28 days. (4)

Because we used the CRT data as a dichotomous variable, the decision of the cut off ≥ 3 seconds for abnormal peripheral perfusion was chosen based on accuracy criteria.

11. The outcome section of the methods more clearly describes the objective, suggests writing something similar in the last paragraph of the introduction.

**Response:** Thank you for the suggestion. We rewrote this information in the Introductions section (p. 4) as follow: “To achieve this goal, this present observational study was designed to investigate the prevalence of peripheral hypoperfusion using CRT and PI between SA-AKI and non-SA-AKI groups, its association with 28 days in-hospital mortality, and correlation with positive fluid balance”.

12. I cannot follow the rationale behind the sample size calculation and the aimed OR of 7.0 between groups, could the authors elaborate?

**Response:** The sample size was calculated based on previous local study (5), which demonstrated an Odds ratio between 8-9 for peripheral hypoperfusion. In this sense, we assumed a smaller Odds ratio, as cited in the methods section as follow:

"The sample size was calculated based on previous local and pilot studies in sepsis.(5) Assuming 28-day mortality around 45-50% in the SA-AKI group and a 40% prevalence of altered peripheral perfusion, estimating a relative risk ratio of 2, we determined that the enrollment of 113 patients in the SA-AKI group would provide a 90% power to detect a mortality difference at an alpha level of 0.05. The previous studies also demonstrated that local non-SA-AKI patients represented approximately 15-20% of septic patients. Considering a peripheral hypoperfusion prevalence of 8% in patients without AKI, 28 patients in the control group were enrolled to have 90% power to reach an odds ratio of 7.0 between groups (p. 9 ).

**Results:**
13. What was the time between admission, study inclusion, and fluid resuscitation?

**Response:** Assessment of patients occurred within 24 h after admission in ICU with the diagnosis of sepsis or within 24 h after the onset of sepsis in patients previously admitted for other causes. Hemodynamic parameters (if available), metabolic, and peripheral variables were measured after fluid resuscitation between 6 and 24 hours of sepsis diagnosis.

We added this information in the Methods section (p 6).

14. The authors could consider either shorten or move tables 1 and 2 to the appendices. They are informative but do not provide the main answers to the main objectives of the study.

### Response: Thanks for your consideration. These tables had been removed from the text and inserted in Supporting information.

14. Did the authors also consider using CRT as a continuous variable?

**Response:** We didn't consider using the CRT as a continuous variable. Although, studies are using the CRT as a continuous variable, the decision of the CRT data's decision as a dichotomous variable was chosen based on accuracy criteria.

15. Could the authors provide more data on the received fluid resuscitation?

**Response:** All selected patients were treated according to our local institution recommendations, which has been adapted from Surviving Sepsis Campaign (SSC) guidelines: 30 ml/kg of ringer lactate or crystalloid fluid were administrated at the discretion of the patients' physicians over the 1 hour of sepsis diagnosis which was continues until lack of response to passive-leg raising (the cutoff value to discriminate fluid responders was an increase in cardiac output of 13%) or no respiratory variations of inferior vena cava diameter (the cutoff was 18%). We revised the Methods section to make it more transparent as to how the analysis was conducted (p. 6).

Because the assessment of peripheral perfusion was made after fluid resuscitation with correction of macro hemodynamic parameters, we didn't collect the volume administered to each patient.Because the assessment of peripheral perfusion was made after fuid resuscitation with correction of macro hemodynamic parameters, we didn’t collected the the amount of volume administrated to each patient.

16. In figure 4, the difference between PI and changes over time is plotted, split for survivors and non-survivors. The title of the figure states the prognostic significance, and a p-value. I do not fully comprehend: does this mean that the changes were prognostic (as there was not a significant alteration) or that all measurements, apart from each other, were consistently different between survivors and nonsurvivors?

**Response:** Figure 4 has three interpretations. The first one shows that there was no interaction between groups and time (p-value not significant). The second one shows an intragroup analysis, where for both survivors and nonsurvivors the PI values didn't change significantly over the first 72 hours (p-value not significant). The third interpretation was an "intergroup analysis," which showed a significant difference in PI values between survivors and nonsurvivors in all three days observed (p-value significant).

This figure answered our objective of analyses the prognostic significance of serial changes in PI values over the first 72 hours in the SA-AKI group. That is the title explanation. We added a legend to clarify the figure (p.11)

**Discussion:**
17. Could the authors specify and clarify what specific pieces of evidence contribute to the support of the use of? CRT? PI? Both? For AKI? Mortality?

**Response:** Our results showed significant differences between survivors and nonsurvivors of SA-AKI patients with PI and CRT methods. These results corroborate studies in non-selected patients with sepsis as prognostic markers for mortality (4–7). Thus, this study brings several new pieces of evidence that can contribute to support its widespread clinical use of CRT and PI methods by nephrologists and non-nephrologists for analyses of mortality.

18. Please change the word humans into something like ´´caregivers´´

**Response:** This has now been corrected (p.12)

19. On page 16, the use of CRT dichotomously or continuously is discussed. Perhaps the authors could add a sensitivity analysis using a different cut off value to see whether this changes the main findings? And here discuss the potential consequences?

**Response:** From the Bayesian perspective, the reliability of a result depends on high pre-test probability. That is why the cutoff point of CRT was chosen based on previous robust studies, and it is precisely for this reason that we believe we have provided more concrete evidence. If we chose the referee's suggestion to make a roc curve to find the cutoff point and perform the causal inferences with the same sample, the result's reliability would potentially reduce.

20. In conclusion, could the authors clarify the sentence: ´´however, the presence of … independently´´. The sentence reads a bit difficult and it is unclear to me what the specific prognostic marker is for (and for what outcome).

**Response:** We rewrote the Conclusion section to make it clearer (p. 16).

References:

1.	Santos D, Quintans J, Quintans Junior L, Santana Filho V, Cunha C, Menezes I. Association between peripheral perfusion, microcircutation and mortality in sepsis: a systematic review. Rev Bras Anestesiol. 2019;69(6):605–21.

2.	International Society of Nephrology. KDIGO Clinical Practice Guideline for Acute Kidney Injury. Kidney Int. 2012;2(1):3.

3.	Ait-Oufella H, Bakker J. Understanding clinical signs of poor tissue perfusion during septic shock. Intensive Care Med. 2016;42(12):2070–2.

4.	Hernández G, Ospina-Tascón GA, Damiani LP, Estenssoro E, Dubin A, Hurtado J, et al. Effect of a Resuscitation Strategy Targeting Peripheral Perfusion Status vs Serum Lactate Levels on 28-Day Mortality among Patients with Septic Shock: The ANDROMEDA-SHOCK Randomized Clinical Trial. JAMA - J Am Med Assoc. 2019;321(7):654–64.

5.	Lara B, Enberg L, Hernandez G, Castro R, Lara B, Bakker J, et al. Capillary refill time during fluid resuscitation in patients with sepsis-related hyperlactatemia at the emergency department is related to mortality. PLoS One. 2017;12(11):e0188548.

6.	Santos DM, et al., MENEZES ICM, et al. Association between peripheral perfusion, microcirculation and mortality in sepsis: a systematic review. Brazilian J Anesthesiol. 2019;69(6):605–21.

7.	Pinto Lima A, Beelen P, Bakker J, Lima AP, et al., Pinto Lima A, et al. Use of a peripheral perfusion index derived from the pulse oximetry signal as a noninvasive indicator of perfusion. Crit Care Med. 2002 Jun;30(6):1210–3.
